# Identifying the most surprising victims of mass extinction events: an example using Late Ordovician brachiopods

**DOI:** 10.1098/rsbl.2017.0400

**Published:** 2017-09-27

**Authors:** Seth Finnegan, Christian M. Ø. Rasmussen, David A. T. Harper

**Affiliations:** 1 Department of Integrative Biology, University of California, Berkeley, CA, USA; 2 Natural History Museum of Denmark, University of Copenhagen, Copenhagen, Denmark; 3 Center for Macroecology, Evolution and Climate, University of Copenhagen, Copenhagen, Denmark; 4 Palaeoecosystems Group, Department of Earth Sciences, Durham University, Durham, UK; 5 Department of Geology, University of Lund, Lund, Sweden

**Keywords:** extinction risk, extinction selectivity, Ordovician, Brachiopoda

## Abstract

Mass extinction events are recognized by increases in extinction rate and magnitude and, often, by changes in the selectivity of extinction. When considering the selective fingerprint of a particular event, not all taxon extinctions are equally informative: some would be expected even under a ‘background’ selectivity regime, whereas others would not and thus require special explanation. When evaluating possible drivers for the extinction event, the latter group is of particular interest. Here, we introduce a simple method for identifying these most surprising victims of extinction events by training models on background extinction intervals and using these models to make per-taxon assessments of ‘expected’ risk during the extinction interval. As an example, we examine brachiopod genus extinctions during the Late Ordovician Mass Extinction and show that extinction of genera in the deep-water ‘*Foliomena* fauna’ was particularly unexpected given preceding Late Ordovician extinction patterns.

## 1. Introduction

Mass extinction events in the fossil record offer the opportunity to study extinction processes during a wide range of biological and environmental perturbations. One of the most striking and informative features of such events is their selectivity. Extinction patterns are rarely consistent with a random ‘field of bullets’ model, but are often strongly selective with respect to ecological, environmental and biogeographic factors [1–4].

Selectivity patterns can be powerful sources of information about the drivers of extinction events, but it is important to consider selectivity patterns during mass extinctions in the context of the intervals of ‘background extinction’ that precede them [5,6]. Extinctions during background intervals are also often highly non-random with respect to a variety of factors [2,5–7]. Consequently, when evaluating a mass extinction event, the critical question is how and to what degree selectivity patterns during the event differ from those observed during previous intervals. Some taxon extinctions are more unexpected—that is, they represent a more surprising departure from background extinction patterns—than others.

Here, we outline a simple method for determining which specific extinctions represent the greatest departures from background patterns. Our approach is to create models of extinction selectivity based on observed extinction/survival patterns during the background extinction intervals leading up to the mass extinction event. We then use these models to predict the expected extinction risk of each taxon during the mass extinction interval based on the risks observed for taxa with similar traits during background intervals.

As an example, we use this approach to examine extinctions of rhynchonelliform (articulate) brachiopods during the Late Ordovician Mass Extinction (LOME), one of the ‘Big 5’ largest extinctions of the past 500 Myr [8,9]. This extinction event occurred in two distinct pulses: a latest Katian (approx. 444.7 Ma) pulse broadly associated with cooling, glaciation and sea-level fall and a mid-Late Hirnantian pulse (at the base of the *persculptus* graptolite biozone) associated with warming, melting of ice sheets and continental flooding [10,11]. The LOME has long been notable for exhibiting relatively little taxonomic selectivity compared with other mass extinction events, but recent analyses of benthic macroinvertebrates have detected significant selectivity related to habitat preference, depth range, abundance and inferred thermal tolerance range [12–17].

## 2. Material and methods

### (a) Database

We base our analyses on a large and taxonomically standardized database of the local stratigraphic ranges of rhynchonelliform brachiopods compiled from the literature and from ongoing research programmes in Durham and Copenhagen [15–17]. Ranges are expressed in terms of the British chronostratigraphic system, which we adapt accordingly: Early Caradoc (Sandbian 1), Mid Caradoc (Sandbian 2), Late Caradoc (Katian 1), Pusgillian (Katian 2), Cautleyan (Katian 3), Rawtheyan (Katian 4) and Hirnantian. These stage subdivisions overlap with, but are not identical to, those of Bergstrom *et al*. [18]. Palaeogeographic coordinates for each local region are based on reconstructions by Cocks & Torsvik [19,20].

Taxonomic revisions were assessed and recommendations implemented to avoid synonyms. Brachiopod genera exhibit strong depth preferences, and benthic assemblage zones (BAs) have long been used as depth indicators in the Early Palaeozoic. Each genus in our dataset was assigned a BA range based on information in the literature, often with reference to associated fauna or lithology. Further details regarding the database are available in previous publications [13,15–17].

### (b) Extinction and risk predictors

Data manipulations and analyses were carried out in the R programming environment [21]. Although it would be desirable to analyse species extinction risk, there is still considerable uncertainty about Ordovician brachiopod taxonomy at the species level. Consequently, we follow many previous analyses of the marine invertebrate fossil record in conducting analyses at the genus level [4,6,22] with the recognition that genera are to some degree artificial constructs. Within each of the seven Late Ordovician intervals analysed, we tabulated several aspects of geographical, bathymetric, environmental and macrostratigraphic distribution for each genus. See ‘Description of predictors’, in the electronic supplementary material, for descriptions of how predictors were calculated and standardized.

### (c) Modelling relationships between predictors and background extinction risk

We examined relationships between the predictors described above and ‘background’ extinction patterns in five pre-extinction intervals (Sandbian 1 and 2, Katian 1–3) and the two pulses of the LOME (Katian 4, Hirnantian). We used stepwise regression (direction = ‘both’) to choose from among the predictors described above and build additive logistic regression models for each interval, in each case selecting the model with the lowest Akaike information criterion. We chose logistic regression as an analytical framework, because it is an established method with well-understood properties that has previously been applied to many analyses of extinction/survival in the fossil record [4,6,7,22].

## 3. Results and discussion

**Figure 1.**
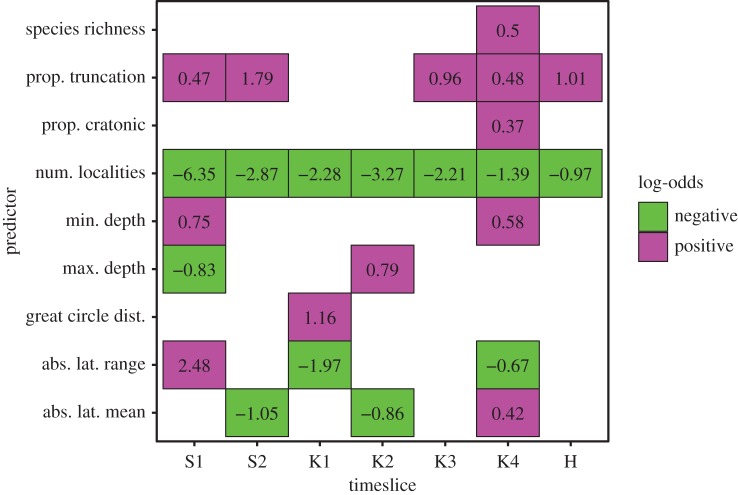
Summary of multiple logistic regression models for different Late Ordovician intervals. Each column represents an interval and each row a predictor. Colours indicate sign of log-odds associated with the predictor in a given interval and text gives the log-odds. Positive log-odds (magenta) indicate that as the predictor value increases marginal extinction risk increases; negative log-odds (green) indicate that as the predictor value increases marginal extinction risk decreases. Only predictors that are significant at the 95% confidence level are plotted. S1: Sandbian 1, S2: Sandbian 2, K1: Katian 1, K2: Katian 2, K3: Katian 3, K4: Katian 4, H: Hirnantian. (Online version in colour.)

Logistic regression coefficients (log-odds ratios) of risk predictors show considerable variability across intervals, demonstrating that the determinants of apparent extinction risk vary both between the mass extinction intervals and the background intervals and among background intervals (figure 1). Consistent with earlier analyses using a different statistical methodology [13], number of localities, proportional stratigraphic truncation, absolute latitudinal range and minimum depth are significantly associated with apparent extinction risk in the latest Katian interval (Katian 4), but of these only number of localities and, to a lesser degree, proportional stratigraphic truncation are consistent predictors of apparent extinction risk during background intervals. We focus the remainder of our discussion on the latest Katian extinction pulse both because it is larger and because it has a more distinct selective signature than the second, Hirnantian pulse [12,13] (figure 1).

**Figure 2.**
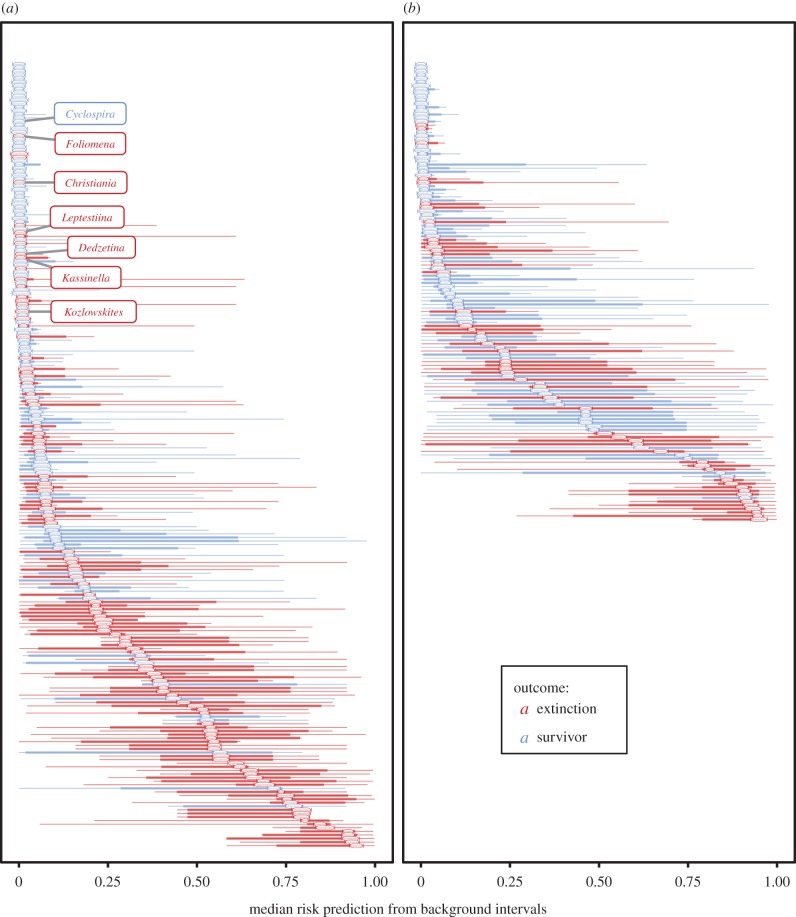
Latest Katian (*a*) and Late Hirnantian (*b*) genera ranked by their median expected extinction risk based on the five background interval models. Thin bars indicate full range of risk predictions from background models; thick bars indicate the range between the second-lowest and second-highest risk predictions. Enlarge figure in online version to read +genus names. Labels indicate core members of the deep-water ‘*Foliomena* fauna’. (Online version in colour.)

Ranking genera present during the Katian 4 and Hirnantian intervals from lowest to highest based on the median risk prediction from the full suite of background models provides an overall measure of expected risk conditioned on prior extinction selectivity patterns (electronic supplementary material, table S1; figure 2). As would be expected considering the consistent importance of predictors such as number of localities and proportional truncation, there is some correspondence between expected risk and observed extinctions: during both intervals most of the genera that go extinct come from the high end of the predicted risk spectrum and most of the genera that survive have low predicted risk. Given that 112 of 219 genera appear to go extinct during Katian 4, the degree of correspondence can be evaluated by comparing observed extinction versus survival with projected extinction versus survival if the 112 genera with the highest expected risk (lower half of figure 2*a*) had gone extinct and the 107 genera with the lowest expected risk (upper half of figure 2*a*) had survived. In total, 79 of 112 observed extinctions fall within this set (true positive percentage = 71%) and 74 of 107 observed survivors are among the projected survivors (true negative percentage = 69%). Of more interest when considering changes in selective regime between the LOME and preceding intervals are the 33 genera with relatively high expected risk that did not go extinct and, especially, the 33 genera that went extinct despite having relatively low expected risk.

No obvious commonalities unite the members of the former group, but conspicuous within the latter group are the members of the ‘*Foliomena* fauna’, a distinctive assemblage of small, thin-shelled genera that were widely distributed in tropical and subtropical seas and inhabited deeper-water (BA 4–6) environments during the Late Ordovician [23,24] (highlighted genera in figure 2*a*). Of seven genera identified as core members of this fauna by Cocks & Rong [25], all but *Cyclospira* disappear in the latest Katian. Simultaneous extinction of graptolite clades associated with oxygen minimum zones [26,27] and proxy evidence for enhanced ventilation of the shelves at this time [28,29] suggest that extinction of the *Foliomena* fauna may represent loss of a distinctive deep-water biotope adapted to low-oxygen conditions widespread during the Katian greenhouse climate state [13] (but see Hammarlund *et al*. [28] for an alternative interpretation). We assessed the probability of observed *Foliomena* fauna extinctions happening by chance given background selectivity patterns and latest Katian extinction rates by randomly drawing 112 genera 10 000 times for each of the five background interval models, in each case using per-genus expected risk estimates from that model as sampling probabilities. The expected probability of extinction of six or more of the core *Foliomena* fauna members is low for all background models, ranging from 0.0133 (Sandbian 1) to <0.0001 (Katian 1 and Katian 3) (electronic supplementary material, figure S1), with an overall probability across all models of 0.0039.

The consistency of number of localities and, to a lesser degree, proportional stratigraphic truncation as predictors of apparent extinction risk (figure 1) may be a genuine signal, as narrowly distributed taxa are at greater risk of extinction under most extinction scenarios, including regression and draining of regional seaways [12,30]. However, this association could also be an artefact of sampling bias. Genera that are only sampled in a single locality are likely to have prematurely truncated stratigraphic ranges, especially if that locality exhibits a hiatus [31,32]. The local range-based nature of our dataset precludes use of occurrence-based approaches to assessing time of extinction [33,34], but to reduce the potential influence of sampling biases we re-ran our analysis after excluding both genera sampled in only a single region and genera sampled in only a single interval. Owing to edge effects Sandbian 1 cannot be included in this analysis, but observed selectivity patterns in the remaining intervals are broadly comparable to those in the full dataset (electronic supplementary material, figure S2). There is a moderately strong positive correlation between median expected risk estimates based on the full dataset and those based on the culled dataset (electronic supplementary material, figure S3), and extinctions of core members of the *Foliomena* fauna remain among the least expected given background extinction patterns (electronic supplementary material, figures S4, and S5). Consequently, we tentatively conclude that, although sampling biases doubtless have an influence on apparent extinction risk patterns, these biases are unlikely to account for the very low expected risk of the *Foliomena* fauna. The extinction of these genera appears to be a genuinely unexpected and potentially informative selective pattern that merits further investigation.

The approach outlined here provides a simple framework for determining which taxa are most likely to have been victims of unusual stresses and which are most likely to have been the expected casualties of ‘normal’ extinction processes. In this example, we have used logistic regression because of its familiarity and simplicity, but a wide variety of statistical methods could be employed. Ecological, physiological and phylogenetic factors could also be considered in assessing expected risk and, when available, taxon occurrence data and data on the spatio-temporal distribution of the stratigraphic record could be incorporated to estimate and reduce the influence of sampling biases. Our approach can be applied to examine other mass extinction events in the fossil record, but could also be used to scan for particularly surprising extinctions that may shed light on extinction processes during background intervals.

## Supplementary Material

Description of potential extinction risk predictors

## Supplementary Material

Figure S1

## Supplementary Material

Figure S2

## Supplementary Material

Figure S3

## Supplementary Material

Figure S4

## Supplementary Material

Figure S5

## Supplementary Material

Table S1

## Data accessibility

Input data (electronic supplementary material, Appendix S1) and R code to replicate our analyses (electronic supplementary material, Appendix S2) are reposited at Data Dryad: http://dx.doi.org/10.5061/dryad.03v3n [35].
