# Supplementary material for: Identifying the most surprising victims of mass extinction events: an example using Late Ordovician brachiopods
Source: Biol Lett. 2017 Sep 27;13(9):20170400. doi: 10.1098/rsbl.2017.0400 (PMC5627174; doi:10.1098/rsbl.2017.0400)
Supplement: Description of potential extinction risk predictors [file rsbl20170400supp1.docx]

**Description of potential extinction risk predictors**

Within each of the seven Late Ordovician intervals analyzed we tabulated several aspects of the geographic, bathymetric, and environmental distribution of each genus, as well as its species richness:

- **Number of Localities**: the number of localities from which the genus is known in that interval.
- **Great Circle Distance**: the maximum great circle distance between localities from which the genus is known in that interval.
- **Absolute Latitudinal Range**: the maximum absolute paleolatitude minus the minimum absolute paleolatitude of localities from which the genus is known in that interval.
- **Mean Absolute Latitude**: the mean absolute paleolatitude of localities from which the genus is known in that interval.
- **Minimum Depth**: the shallowest Benthic Assemblage in which the genus occurs.
- **Maximum Depth**: the deepest Benthic Assemblage in which the genus occurs.
- **Species Richness**: the number of named species assigned to the genus during that interval. Genera lacking named species (e.g., all occurrences in that interval were recorded as “sp.”) were assigned a species richness of 1.
- **Species Richness**: the number of named species assigned to the genus during that interval.
- **Proportion Cratonic**: the proportion of all localities occupied by the genus during that interval that were located in cratonic seaways

We categorized each locality in our database as stratigraphically continuous in a given interval if at least one brachiopod genus occurred at that locality in the immediately succeeding interval, and stratigraphically discontinuous (i.e., truncated) if no brachiopod genera are documented from that locality in the succeeding interval. Discontinuous sections are assumed to represent failures of preservation (lack of appropriate rock record and/or collection effort). To determine whether such discontinuities were an important predictor of apparent extinction risk, we also tallied:

- **Proportional Truncation**: the proportion of all localities occupied by the genus in that interval that are truncated before the next interval (that is, they are represented only by a hiatus in the succeeding interval)

All predictors were standardized prior to analysis, using the scale()(z-score) function for unbounded predictors and the qnorm() function for bounded predictors.
