# Supplementary material for: Identifying the most surprising victims of mass extinction events: an example using Late Ordovician brachiopods
Source: Biol Lett. 2017 Sep 27;13(9):20170400. doi: 10.1098/rsbl.2017.0400 (PMC5627174; doi:10.1098/rsbl.2017.0400)
Supplement: Figure S1 [file rsbl20170400supp2.pdf]

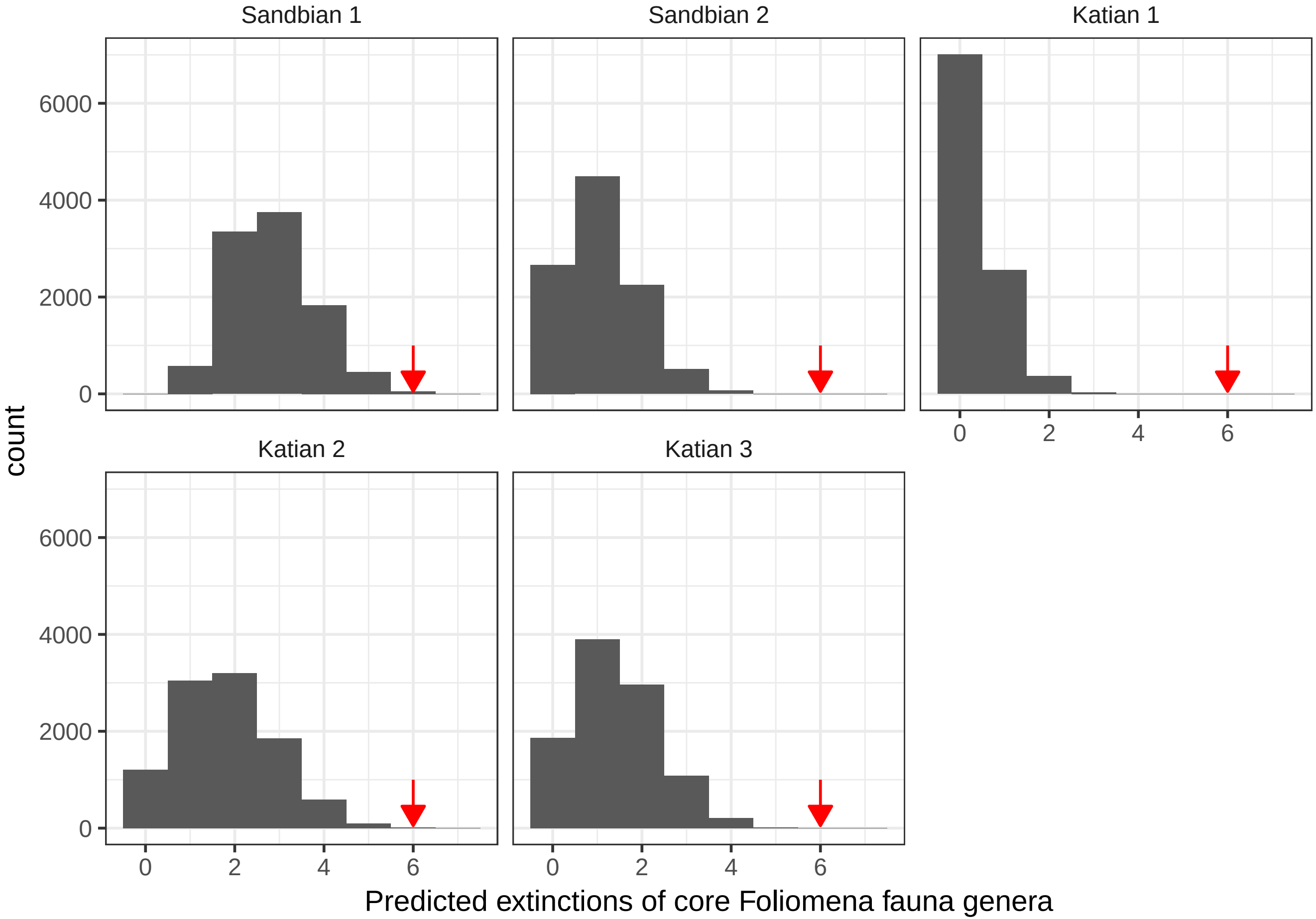
Sandbian 1
Sandbian 2
Katian 1
Katian 2
Katian 3
count
6000
4000
2000
0
0
2
4
6
Predicted extinctions of core Foliomena fauna genera
