# Supplementary figures and images for: Identifying the most surprising victims of mass extinction events: an example using Late Ordovician brachiopods
Source: Biol Lett. 2017 Sep 27;13(9):20170400. doi: 10.1098/rsbl.2017.0400 (PMC5627174; doi:10.1098/rsbl.2017.0400)

Predictor

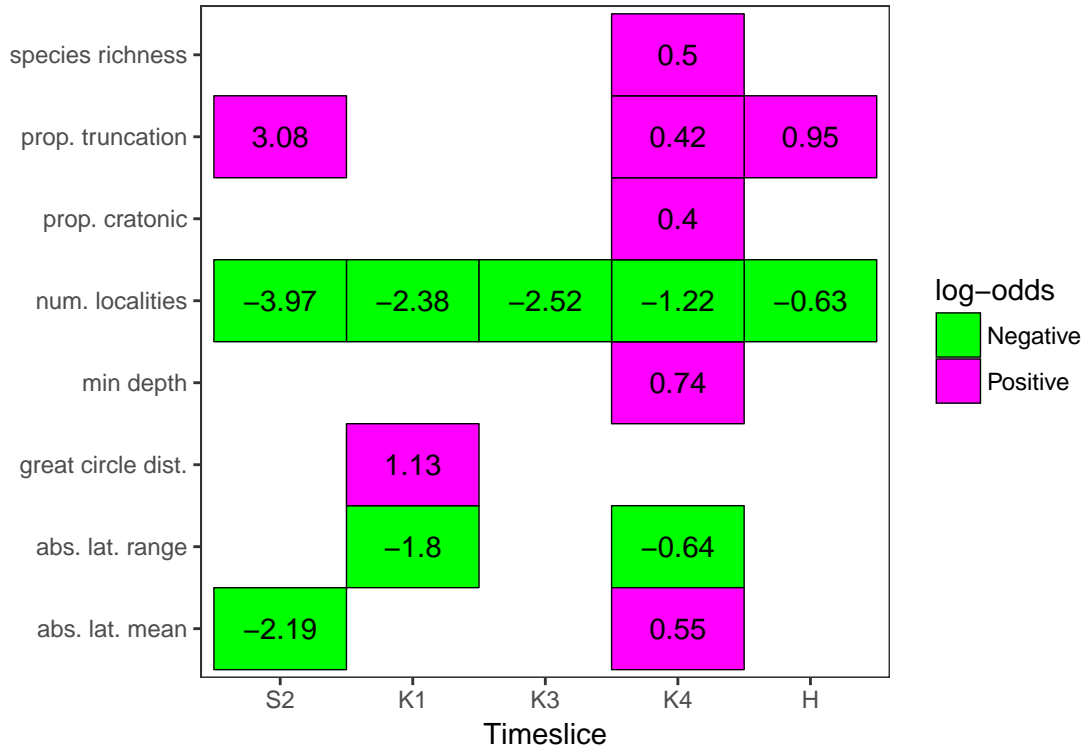

Supplement: Figure S2 [file rsbl20170400supp3.pdf]

A. Latest Katian

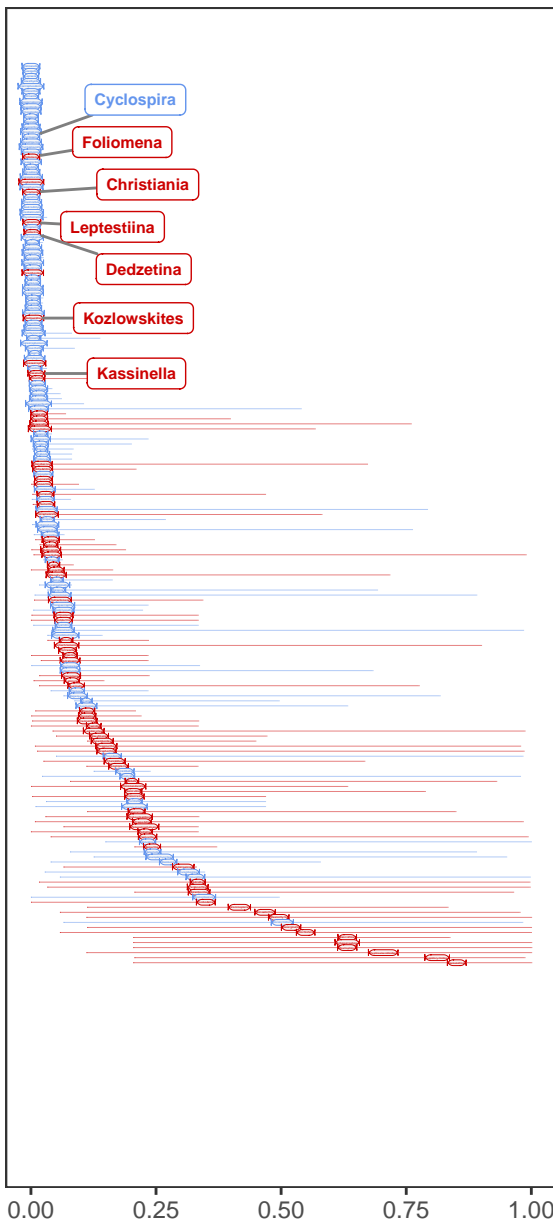

B. Hirnantian

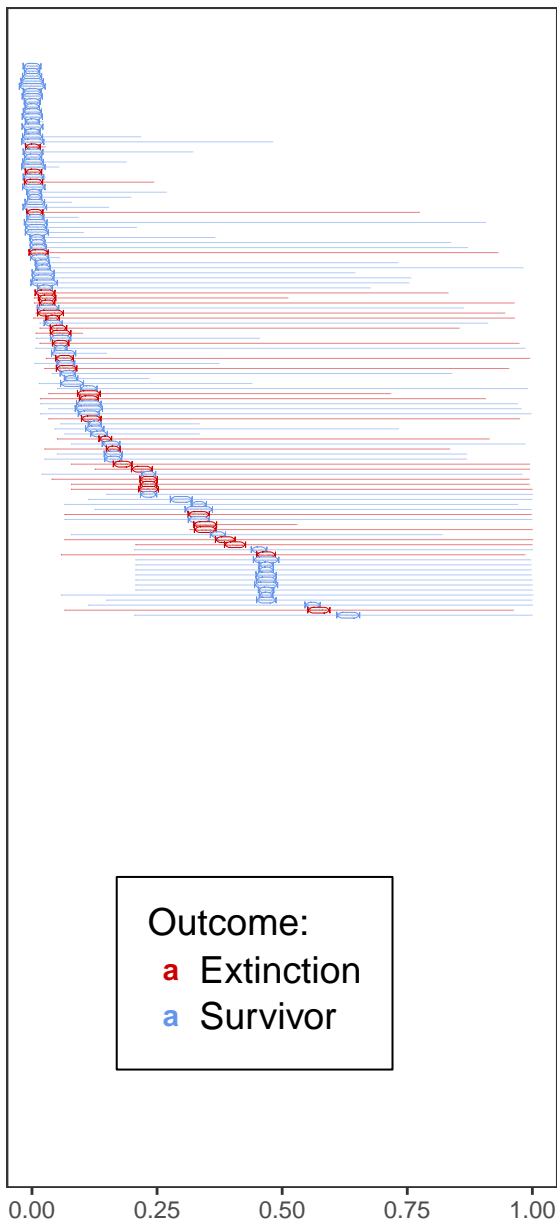

Median risk prediction from background intervals

Supplement: Figure S3 [file rsbl20170400supp4.pdf]

A. Latest Katian

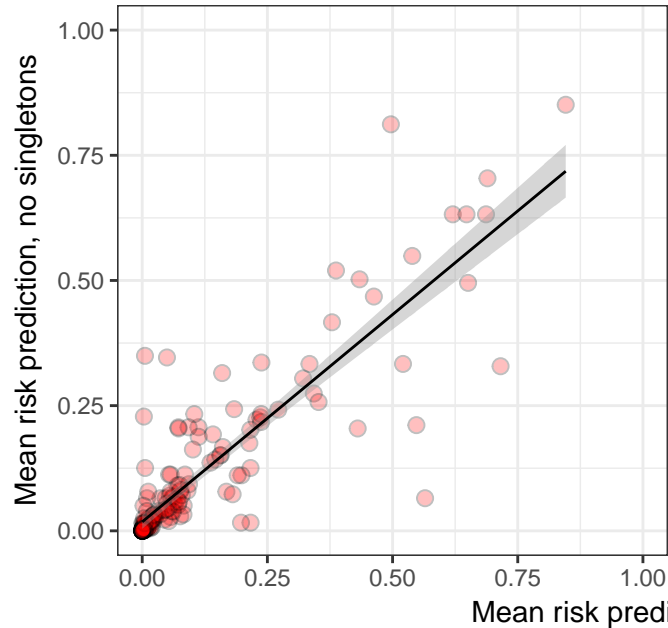

B. Hirnantian

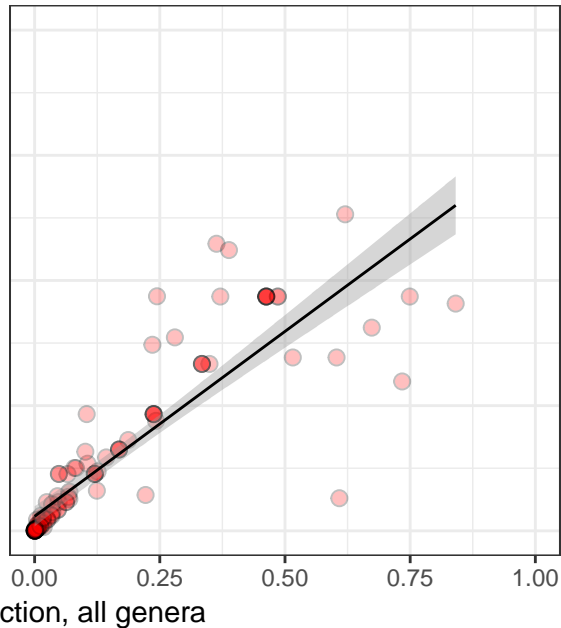

Supplement: Figure S4 [file rsbl20170400supp5.pdf]

Sandbian 2

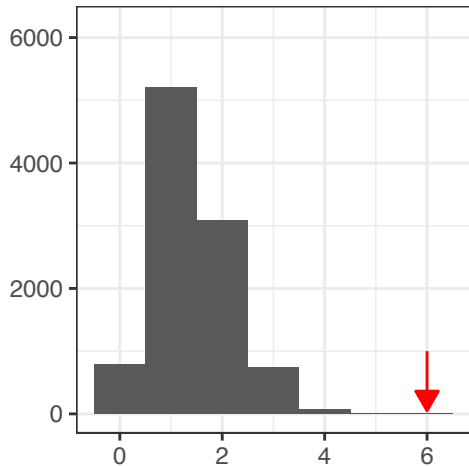

Katian 1

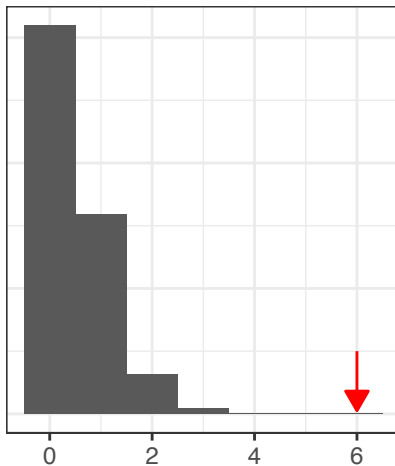

Katian 3

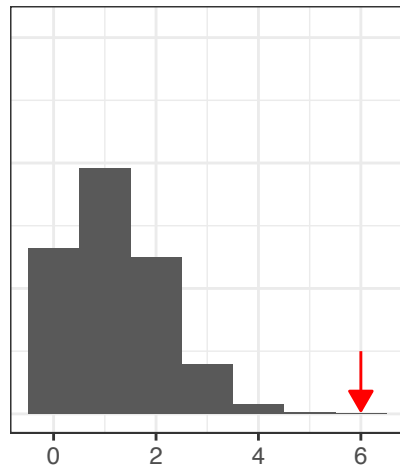

Predicted extinctions of core Foliomena fauna genera

Supplement: Figure S5 [file rsbl20170400supp6.pdf]
